# Gut Microbiota and Primary Liver Cancer: Mendelian Randomization and Network Pharmacology Used to Predict Potential Therapeutic Intervention With Traditional Chinese Medicine

**DOI:** 10.1155/ijog/8161619

**Published:** 2026-04-30

**Authors:** Qingliang Chen, Lin Jin, Suping Ding, Lishan Ding, Liutong Zhang, Shujuan Zuo, Wenle Fu, Pengchao Zhan

**Affiliations:** ^1^ Department of Radioactive Intervention Department, Henan No.3 Provincial People’s Hospital, Zhengzhou, Henan, China; ^2^ Department of Pharmacy, Lijiang People’s Hospital, Lijiang, Yunnan, China; ^3^ School of Rehabilitation, Henan University of Chinese Medicine, Zhengzhou, Henan, China, hactcm.edu.cn; ^4^ Department of Hematology and Oncology, The First Affiliated Hospital of Henan University of Traditional Chinese Medicine, Zhengzhou, Henan, China, hactcm.edu.cn

**Keywords:** causal effect, gut microbiota, Mendelian randomization, network pharmacology, primary liver cancer

## Abstract

**Objective:**

The objective of this study is to employ Mendelian randomization (MR) to screen for gut microbiota (GM) exhibiting causal genetic effects on primary liver cancer (PLC) development and to predict promising traditional Chinese medicine (TCM) candidates capable of intervening in PLC by modulating the GM.

**Methods:**

Summary statistics from genome‐wide association studies (GWASs) examining the relationship between GM and PLC were retrieved from the IEU OpenGWAS database. MR analysis was conducted using the two‐sample MR package in R, employing the inverse variance weighted (IVW) method as the primary approach for assessing genetic causal effects. Functional enrichment analysis was performed on genes proximal to the instrumental variables (IVs) to investigate the signaling pathways through which the implicated microbiota may contribute to PLC pathogenesis. The CTD and Coremine Medical databases were combined to predict TCM compounds potentially regulating the genes proximal to the IVs.

**Result:**

The MR analysis identified nine taxonomic groups of GM exhibiting genetically predicted causal effects on PLC development. Among these, the Family XI (OR = 0.680, 95% CI: 0.469–0.985, *p* = 0.041), the genus *Anaerotruncus* (OR = 0.446, 95% CI: 0.228–0.873, *p* = 0.018), the genus *Coprococcus* 2 (OR = 0.440, 95% CI: 0.208–0.929, *p* = 0.031), the genus *Escherichia Shigella* (OR = 0.430, 95% CI: 0.216–0.857, *p* = 0.016), and the order Lactobacillales (OR = 0.517, 95% CI: 0.280–0.955, *p* = 0.035) were associated with a reduced risk of PLC. Conversely, the genus *Barnesiella* (OR = 2.594, 95% CI: 1.388–4.849, *p* = 0.003), the genus *Catenibacterium* (OR = 1.749, 95% CI: 1.003–3.048, *p* = 0.049), the genus *Ruminococcus* 2 (OR = 1.808, 95% CI: 1.066–3.068, *p* = 0.028), and Mollicutes RF9 (OR = 1.707, 95% CI: 1.010–2.884, *p* = 0.046) were associated with an elevated risk of PLC. The most frequently represented Chinese medicinal herbs predominantly include *Camellia sinensis* root, *Curcuma longa* L., *Salvia miltiorrhiza*, *Triticum aestivum* L., *Panax ginseng* C. A. Meyer, *Panax notoginseng* F.H. Chen, Radix Curcumae, *Aucklandia lappa*, *Scutellaria baicalensis*, and *Zingiber officinale* Rose., among others. GO enrichment analysis revealed that these genes were significantly enriched in biological processes including positive regulation of the ERK1/ERK2 signaling pathway, posttranslational protein modification (such as deglutamylation), synaptic vesicle exocytosis, and urea transport. KEGG pathway enrichment analysis demonstrated predominant enrichment of these genes in the neurotrophin signaling pathway and the cAMP signaling pathway.

**Conclusion:**

This study provides novel insights for developing GM‐based TCM strategies for PLC prevention and treatment.

## 1. Introduction

Primary liver cancer (PLC) represents one of the leading causes of cancer‐related mortality worldwide. It is estimated that approximately 800,000 deaths occur annually due to this disease, with projections indicating that fatalities will exceed one million by 2030 [1–3]. Surgical resection serves as the primary therapeutic approach for early‐stage liver cancer; however, it carries significant risks of complications including liver failure, intra‐abdominal infections, and pleural effusion, which pose serious threats to patient health [4]. Previous research has identified chronic alcohol consumption—via induction of chronic liver injury and cirrhosis—as well as metabolic dysregulation and chronic inflammation associated with obesity and overweight, along with the fibrosis–cirrhosis pathway in nonalcoholic steatohepatitis (NASH) progression, as significant drivers of PLC development [5, 6]. In recent years, investigations have begun to explore other potential oncogenic factors. Notably, GM dysbiosis, which modulates hepatic immunity and metabolism through the gut–liver axis, has broadened our understanding of liver cancer etiology, introducing a novel dimension to this field [7]. With the establishment of the 2020–2025 Dietary Guidelines for Americans highlighting nutritional science goals, the critical role of the microbiome in health and disease pathogenesis has garnered significant attention [8]. The human body functions as a vast metaorganism, harboring immense microbial communities residing in niches such as the gut, oral cavity, respiratory tract, and skin [9]. Human health is intrinsically linked to these microbiotas through a symbiotic relationship; dysbiosis in any niche or microbial taxon can trigger a transition from health to disease [10, 11]. While microbes are now recognized as significant environmental carcinogenic factors, the ubiquitous yet heterogeneous distribution and complex taxonomic composition of the human microbiome present a major challenge: Identifying potential procarcinogenic microbial signatures within this diversity is central to elucidating the association between the microbiome and PLC.

Traditional Chinese medicine (TCM) emphasizes the physiological and pathological connections between the liver and the intestines, a holistic perspective that has been scientifically validated in modern medicine through the concept of the “gut–liver axis” (a GM–immune–metabolic network). Modern research has established the core concept of the “gut–liver axis,” highlighting the intricate interaction between the liver and the large intestine via bidirectional communication pathways [12]. Gut dysbiosis can impair intestinal barrier function, allowing bacterial metabolites such as lipopolysaccharide (LPS) and secondary bile acids to translocate into the liver via the portal vein. This process activates Toll‐like receptor (TLR4/9) signaling pathways, triggering chronic inflammation, fibrosis, and hepatocellular carcinoma [13, 14]. Further MR studies have confirmed that there is a bidirectional causal relationship between the GM and biliary tumors, and changes in immune cell phenotypes play a key mediating role in this process [15, 16]. Conversely, bile acids secreted by the liver modulate GM composition, barrier integrity, and immune responses [17]. It has been found that a TCM formulation containing three natural medicinal agents significantly enhances the GM profile, maintains intestinal mucosal barrier integrity, boosts immune function, improves bile acid metabolism, and mitigates adverse effects such as nausea, vomiting, and diarrhea in patients with PLC [18]. Furthermore, studies employing probiotic interventions in murine models of liver cancer based on the gut–liver axis have demonstrated that probiotics significantly reduce levels of IL‐17 and Th17 cells. Additionally, they suppress tumor angiogenesis, reduce tumor volume, and enhance the therapeutic efficacy against liver cancer by modulating the GM. This enhancement occurs through mechanisms including strengthening intestinal barrier integrity, modulating immune responses, and improving secondary bile acid metabolism [19]. Relevant studies have revealed that, compared to healthy individuals, patients with PLC exhibit a reduced abundance of the phylum Firmicutes at the phylum level. At the genus level, significant decreases in the abundance of microbial genera such as *Blautia* and *Bacteroides* were observed, while the genus *Streptococcus* showed a marked increase in abundance [20]. Modulating the GM composition can partially reverse immune evasion in PLC and inhibit tumor progression [21, 22]. Collectively, these findings indicate that the GM plays a crucial role in the pathogenesis and progression of PLC.

Mendelian randomization (MR) is a causal inference method that utilizes genetic variants as IVs and is widely applied in epidemiology [23–25]. Its fundamental principle leverages the influence of naturally randomized genetic variation on phenotypes to infer the effects of biological factors on disease outcomes [26]. Consequently, MR enhances the accuracy of causal effect estimates and ensures its advantage in inferring causality. Genome‐wide association studies (GWASs), a cornerstone methodology in statistical genetics, posit that traits are genetically determined [27]. GWAS identifies genomic variants significantly associated with traits through genome‐wide genotyping. Building upon this, the present study is aimed at utilizing GWAS data on GM and PLC to identify gut microbial taxa exhibiting significant causal relationships with PLC development via the MR approach. Furthermore, it seeks to explore TCMs with potential regulatory effects on gut microbiota‐mediated PLC pathogenesis. This investigation holds significant clinical implications for developing targeted preventive and therapeutic strategies against PLC.

## 2. Materials and Methods

### 2.1. Study Design

**Figure 1:**
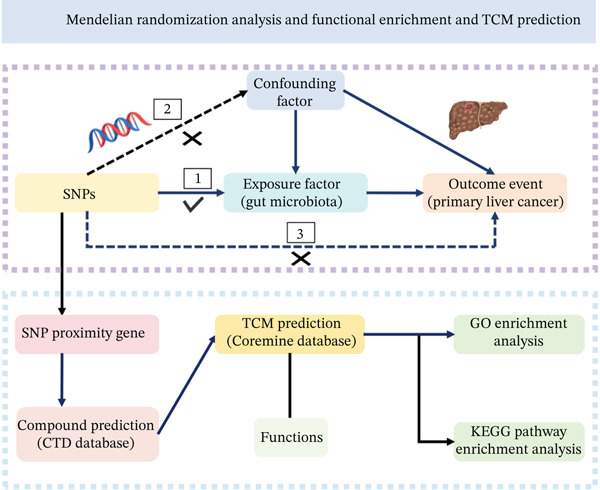
Study design of MR analysis and functional enrichment and TCM prediction.

A two‐sample MR study was designed to estimate the potential causal link between GM and PLC. MR studies were required to satisfy three core assumptions [28, 29]: (1) IVs must be significantly associated with the exposure (GM); (2) IVs must not be associated with any confounding factors; (3) IVs must influence the outcome PLC exclusively through the exposure. Subsequently, genes proximal to the identified IVs were obtained. Functional enrichment analysis was then conducted to investigate key biological pathways through which the GM mediates PLC development and to predict potential TCM compounds capable of modulating these pathways. The overall study workflow was illustrated in Figure 1.

### 2.2. GWAS Summary Statistics

The GWAS summary data of GM were sourced from the IEU OpenGWAS database (https://gwas.mrcieu.ac.uk/). Utilizing the ao function of the TwoSampleMR software package in R, we extracted 418 GWAS summary datasets for 211 GM traits from the IEU OpenGWAS database. Data for PLC were obtained from the FinnGen database’s GWAS summary statistics. The study comprised 500 PLC cases and 314,193 healthy control individuals.

### 2.3. Ethical Approval

The MR analysis in this study utilized publicly available GWAS summary statistics. All original data underlying these summary statistics have been approved by the respective institutional review boards in the source studies. Thus, the requirement for ethical approval was waived for this study.

### 2.4. Instrumental Variable Selection

This study uses SNPs from GWAS data as IVs, screened through a standardized process: Firstly, SNPs significantly associated with the target trait (GM trait) at *p* < 5 × 10^−6^ were extracted from the GWAS data. Linkage disequilibrium (LD) clumping (*r* ^2^ < 0.001, window size = 10,000 kb) was then applied to remove redundant genetic signals. Subsequently, variants with a minor allele frequency (MAF) < 0.01 were excluded to filter out low‐frequency variants. SNPs associated with potential confounders were further removed using PhenoScanner V2 (*p* < 1.0 × 10^−5^, *r* ^2^ ≥ 0.8, Build 37/GRCh37) to mitigate horizontal pleiotropy bias. Subsequently, the *F*‐statistic (*F* = *β* ^²^/SE^²^) was calculated for each SNP to exclude weak instruments (*F* < 10), while palindromic sequences were identified and excluded through base complementary pairing analysis. This rigorous process resulted in a robust set of SNPs satisfying the three core IV assumptions, thereby ensuring the biological validity and statistical power of the causal inference.

The specific calculation formula of *F* value is as follows:

F=N−K−1×R2÷1−R2÷K,

R2=2×1−MAF×MAF×β2,

where *N* is the sample size in the exposure dataset, *R* ^2^ represents the proportion of variance explained by the SNPs in the exposure dataset, MAF denotes the MAF, and *β* is the allele effect size.

### 2.5. Statistical Analysis

This study primarily employed the IVW method to assess genetic causal effects [30], with supplementary analyses using MR‐Egger, simple mode, weighted median, and weighted mode approaches to evaluate the robustness of IVW results [31]. First, Wald ratios were calculated for each SNP and subsequently combined through weighted integration to derive associations between genetically predicted GM and the risk of PLC. Given the binary nature of the outcome (risk of PLC), odds ratios (ORs) with 95% confidence intervals (CIs) were utilized to present potential causal relationships between GM and malignant tumors. Furthermore, this study conducted the following sensitivity analyses [32]: (1) genetic heterogeneity assessment: Cochran’s *Q* statistic was calculated using the IVW and MR‐Egger methods to evaluate the dispersion of effect sizes across genetic instrumental variants. When the test reached the significance threshold (*p* < 0.05), a random‐effects model was employed to correct for heterogeneity bias. (2) Horizontal pleiotropy detection: The presence of significant pleiotropy was determined based on the statistical significance (*p* < 0.05) of the intercept term in MR‐Egger regression. Causal effect estimates under this condition were interpreted with caution. (3) Exclusion of palindromic SNPs: SNPs exhibiting palindromic characteristics (with ambiguous strand orientation due to complementary base pairing) were removed. (4) Screening for variants associated with potential confounders: Genetic variants showing genome‐wide significant associations (*p* < 5 × 10^−8^) with potential confounding phenotypes were excluded, utilizing publicly available phenotype association data from the GWAS Catalog (https://www.ebi.ac.uk/gwas/). (5) Leave‐one‐out cross‐validation: The IVW regression was iteratively performed by excluding each individual SNP to assess the potential influence of single genetic markers on the overall results. (6) MR‐Pleiotropy RESidual Sum and Outlier (MR‐PRESSO) outlier detection and correction: The MR‐PRESSO algorithm was applied to detect and correct for outliers, identifying aberrant IVs through its global test. A *p* value < 0.05 was considered statistically significant. All statistical analyses were performed using R Version 4.3.2.

### 2.6. IVs Near Gene Function Enrichment and TCM Prediction

Firstly, the R packages vautils and dplyr were employed to identify proximal genes to the IVs based on SNP identifiers (IDs), along with their corresponding chromosome sequences and positions. Subsequently, these genes were queried against the Comparative Toxicogenomics Database (CTD; https://ctdbase.org/) to identify chemicals exerting regulatory effects on them. Chemicals demonstrating substantial literature support were selected. Using the Coremine Medical database (https://coremine.com/medical/), TCMs significantly associated with these identified chemicals were retrieved, applying a significance threshold of *p* < 0.05 for subsequent efficacy analysis. Finally, Gene Ontology (GO) and Kyoto Encyclopedia of Genes and Genomes (KEGG) pathway enrichment analyses were performed on the proximal genes to the IVs utilizing the Metascape platform (https://metascape.org/gp/index.html).

## 3. Results

### 3.1. The MR Analysis Results

**Figure 2:**
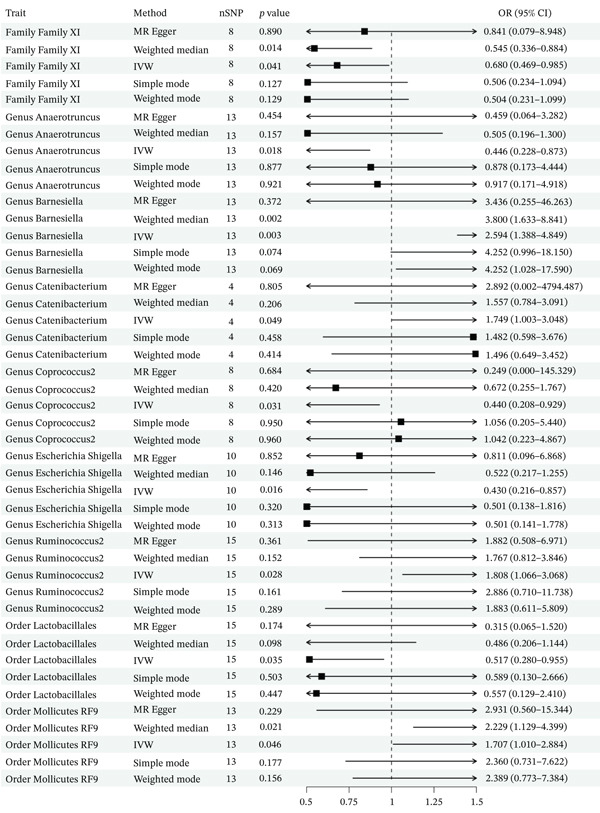
MR forest graph analysis results. From left to right, exposure factors, study methods, IVs, *p* value (*p* value < 0.05 is defined as a positive result), and OR value (OR value > 1 is a risk factor and < 1 is a protective factor) are represented.

**Figure 3:**
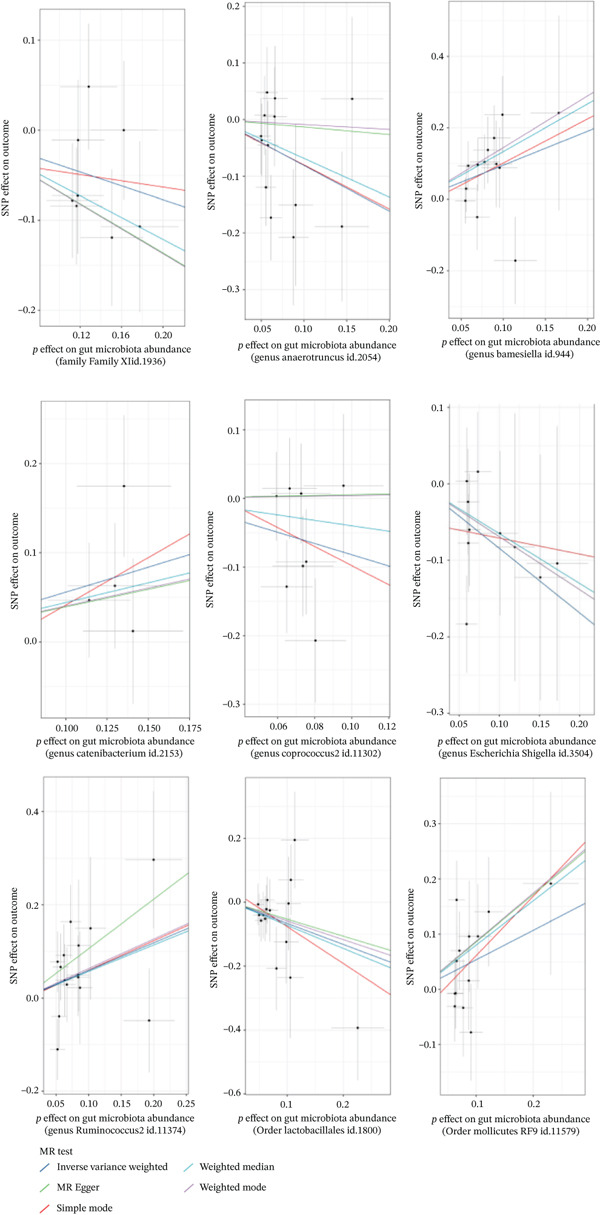
MR scatter plot analysis results.

A total of 2092 SNPs were yielded from 211 GM traits. We identified no SNPs associated with confounding factors; subsequently, these SNPs were included in the subsequent MR analysis. In the MR analysis, 99 SNPs served as IVs, all exhibiting *F*‐statistics greater than 15, indicating no evidence of weak instruments. The IVW method revealed nine GM taxa with significant genetic causal effects on PLC risk. Among these, five were associated with a significantly reduced risk of PLC, while four were associated with a significantly increased risk. As shown in Table 1, the Family XI id.1936 (OR = 0.680, 95% CI: 0.469–0.985, *p* = 0.041), the genus *Anaerotruncus* (OR = 0.446, 95% CI: 0.228–0.873, *p* = 0.018), the genus *Coprococcus* 2 (OR = 0.440, 95% CI: 0.208–0.929, *p* = 0.031), the genus *Escherichia Shigella* (OR = 0.430, 95% CI: 0.216–0.857, *p* = 0.016), and the order Lactobacillales (OR = 0.517, 95% CI: 0.280–0.955, *p* = 0.035) could significantly reduce the risk of PLC. Conversely, the genus *Barnesiella* (OR = 2.594, 95% CI: 1.388–4.849, *p* = 0.003), the genus *Catenibacterium* (OR = 1.749, 95% CI: 1.003–3.048, *p* = 0.049), the genus *Ruminococcus* 2 (OR = 1.808, 95% CI: 1.066–3.068, *p* = 0.028), and the order Mollicutes RF9 (OR = 1.707, 95% CI: 1.010–2.884, *p* = 0.046) could significantly increase the risk of PLC. The direction of effects in the MR‐Egger analysis was consistent with that of the IVW results for all GM, supporting the robustness of the IVW findings. The forest plot and scatter plot for the analysis are presented in Figures 2 and 3, respectively.

**Table 1:** Positive results of MR analysis of GM and PLC.

Exposure	Method	nSNP	*p*	OR	or_lci95	or_uci95
Family Family XI id.1936	Weighted median	8	0.014	0.545	0.336	0.884
Family Family XI id.1936	Inverse variance weighted	8	0.041	0.680	0.469	0.985
Family Family XI id.1936	Simple mode	8	0.127	0.506	0.234	1.094
Family Family XI id.1936	Weighted mode	8	0.129	0.504	0.231	1.099
Family Family XI id.1936	MR‐Egger	8	0.890	0.841	0.079	8.948
Genus *Anaerotruncus* id.2054	Inverse variance weighted	13	0.018	0.446	0.228	0.873
Genus *Anaerotruncus* id.2054	Weighted median	13	0.157	0.505	0.196	1.300
Genus *Anaerotruncus* id.2054	MR‐Egger	13	0.454	0.459	0.064	3.282
Genus *Anaerotruncus* id.2054	Simple mode	13	0.877	0.878	0.173	4.444
Genus *Anaerotruncus* id.2054	Weighted mode	13	0.921	0.917	0.171	4.918
Genus *Barnesiella* id.944	Weighted median	13	0.002	3.800	1.633	8.841
Genus *Barnesiella* id.944	Inverse variance weighted	13	0.003	2.594	1.388	4.849
Genus *Barnesiella* id.944	Weighted mode	13	0.069	4.252	1.028	17.590
Genus *Barnesiella* id.944	Simple mode	13	0.074	4.252	0.996	18.150
Genus *Barnesiella* id.944	MR‐Egger	13	0.372	3.436	0.255	46.263
Genus *Catenibacterium* id.2153	Inverse variance weighted	4	0.049	1.749	1.003	3.048
Genus *Catenibacterium* id.2153	Weighted median	4	0.206	1.557	0.784	3.091
Genus *Catenibacterium* id.2153	Weighted mode	4	0.414	1.496	0.649	3.452
Genus *Catenibacterium* id.2153	Simple mode	4	0.458	1.482	0.598	3.676
Genus *Catenibacterium* id.2153	MR‐Egger	4	0.805	2.892	0.002	4794.487
Genus *Coprococcus* 2 id.11302	Inverse variance weighted	8	0.031	0.440	0.208	0.929
Genus *Coprococcus* 2 id.11302	Weighted median	8	0.420	0.672	0.255	1.767
Genus *Coprococcus* 2 id.11302	MR‐Egger	8	0.684	0.249	0.000	145.329
Genus *Coprococcus* 2 id.11302	Simple mode	8	0.950	1.056	0.205	5.440
Genus *Coprococcus* 2 id.11302	Weighted mode	8	0.960	1.042	0.223	4.867
Genus *Escherichia Shigella* id.3504	Inverse variance weighted	10	0.016	0.430	0.216	0.857
Genus *Escherichia Shigella* id.3504	Weighted median	10	0.146	0.522	0.217	1.255
Genus *Escherichia Shigella* id.3504	Weighted mode	10	0.313	0.501	0.141	1.778
Genus *Escherichia Shigella* id.3504	Simple mode	10	0.320	0.501	0.138	1.816
Genus *Escherichia Shigella* id.3504	MR‐Egger	10	0.852	0.811	0.096	6.868
Genus *Ruminococcus* 2 id.11374	Inverse variance weighted	15	0.028	1.808	1.066	3.068
Genus *Ruminococcus* 2 id.11374	Weighted median	15	0.152	1.767	0.812	3.846
Genus *Ruminococcus* 2 id.11374	Simple mode	15	0.161	2.886	0.710	11.738
Genus *Ruminococcus* 2 id.11374	Weighted mode	15	0.289	1.883	0.611	5.809
Genus *Ruminococcus* 2 id.11374	MR‐Egger	15	0.361	1.882	0.508	6.971
Order Lactobacillales id.1800	Inverse variance weighted	15	0.035	0.517	0.280	0.955
Order Lactobacillales id.1800	Weighted median	15	0.098	0.486	0.206	1.144
Order Lactobacillales id.1800	MR‐Egger	15	0.174	0.315	0.065	1.520
Order Lactobacillales id.1800	Weighted mode	15	0.447	0.557	0.129	2.410
Order Lactobacillales id.1800	Simple mode	15	0.503	0.589	0.130	2.666
Order Mollicutes RF9 id.11579	Weighted median	13	0.021	2.229	1.129	4.399
Order Mollicutes RF9 id.11579	Inverse variance weighted	13	0.046	1.707	1.010	2.884
Order Mollicutes RF9 id.11579	Weighted mode	13	0.156	2.389	0.773	7.384
Order Mollicutes RF9 id.11579	Simple mode	13	0.177	2.360	0.731	7.622
Order Mollicutes RF9 id.11579	MR‐Egger	13	0.229	2.931	0.560	15.344

### 3.2. Quality Control Results of MR Analysis

**Figure 4:**
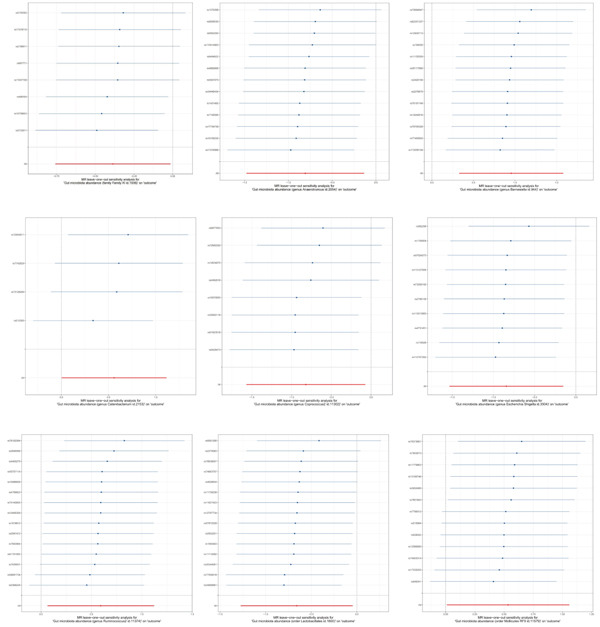
MR analysis of the causal relationship between GM and PLC “leave‐one‐out” map.

As presented in Table 2, the MR‐Egger intercept test indicated no evidence of horizontal pleiotropy for the genetically predicted causal associations between all GM and PLC (*p* > 0.05) [31]. Cochran’s *Q* test revealed no significant heterogeneity among the IVs (SNPs) (*p* > 0.05); the observed heterogeneity was deemed acceptable within the MR framework and did not compromise the causal effect estimates. Subsequently, leave‐one‐out sensitivity analysis was performed to assess the influence of individual SNPs on the overall causal estimate. As illustrated in Figure 4, the causal association remained statistically significant, and the direction of effect was consistent upon systematic removal of each individual SNP, demonstrating the robustness of the findings.

**Table 2:** Quality control results of MR analysis.

GM	Number of SNPs	MR‐Egger intercept	Cochran’s *Q*
Family Family XI	8	0.865	0.717
Genus *Anaerotruncus*	13	0.976	0.578
Genus *Barnesiella*	13	0.831	0.483
Genus *Catenibacterium*	4	0.906	0.511
Genus *Coprococcus* 2	8	0.866	0.398
Genus *Escherichia Shigella*	10	0.556	0.693
Genus *Ruminococcus* 2	15	0.948	0.472
Order Lactobacillales	15	0.515	0.730
Order Mollicutes RF9	13	0.514	0.673

### 3.3. Functional Enrichment Analysis of Instrumental Variable‐Adjacent Genes and Prediction of Potential TCMs

**Figure 5:**
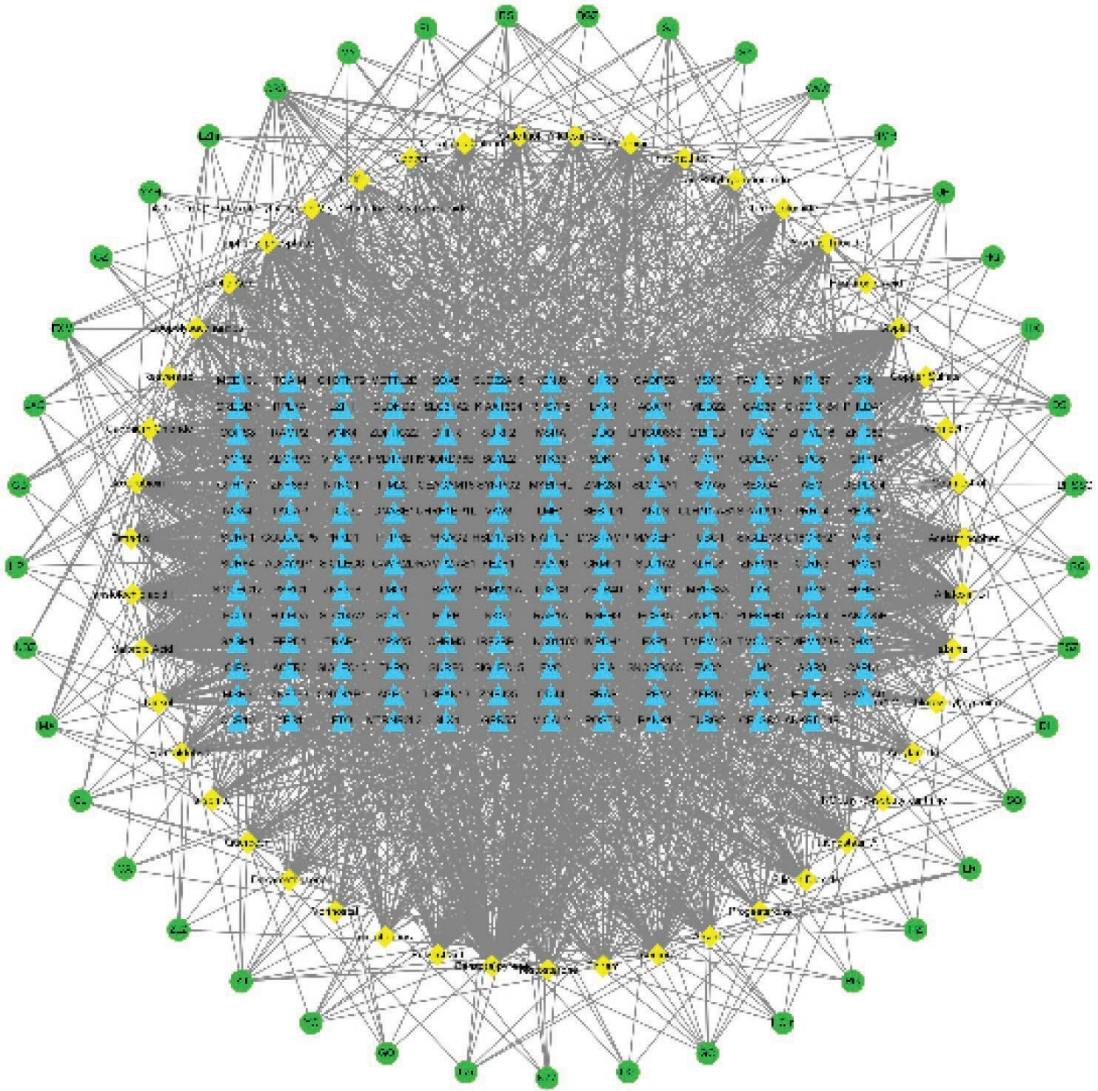
Mapping network diagram of “gene–chemical composition–TCM.”

**Figure 6:**
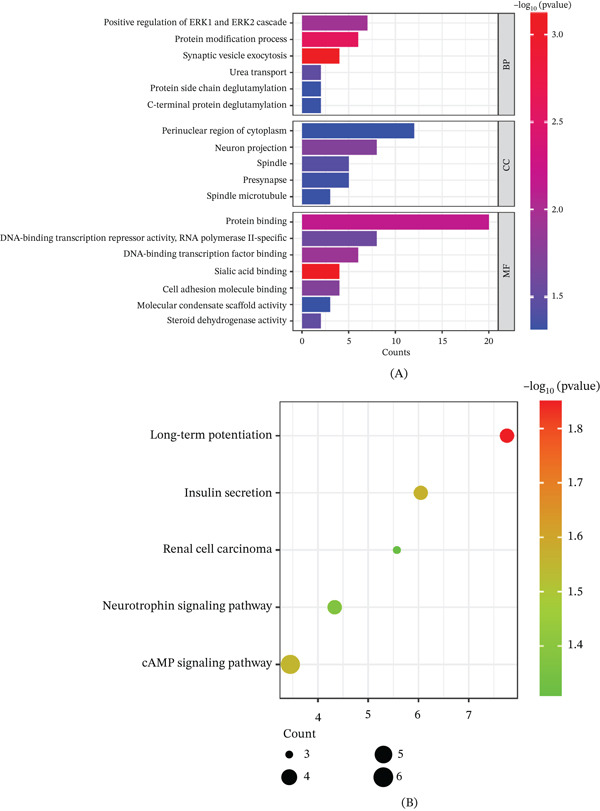
Functional enrichment analysis.

Based on SNP IDs and their chromosomal locations, 229 genes corresponding to 99 SNPs were identified. These genes were submitted to the CTD, yielding 232 chemical compounds including but not limited to cyclosporine, methotrexate, sunitinib, and retinoic acid. Subsequent interrogation of the Coremine database identified 928 medicinal herbs significantly associated with these 232 chemical constituents. Using Cytoscape, the Top 150 nodes by degree centrality within the “gene–chemical composition–TCMs” association network were visualized, as shown in Figure 5. The representative TCM with the highest mapping frequency included *Camellia sinensis* root, *Curcuma longa* L., *Salvia miltiorrhiza*, *Triticum aestivum* L., *Panax ginseng* C. A. Meyer, *Panax notoginseng* F.H. Chen, Radix Curcumae, *Aucklandia lappa*, *Scutellaria baicalensis*, and *Zingiber officinale* Rose., among others. Finally, functional enrichment analysis was performed on genes associated with both the predicted TCMs and their chemical compositions. GO enrichment analysis demonstrated significant enrichment of these genes in biological processes including positive regulation of the ERK1/ERK2 signaling pathway, posttranslational protein modifications such as deglutamylation, synaptic vesicle exocytosis, and urea transport (Figure 6A). Additionally, they were enriched in cellular components such as neuron projection and spindle apparatus and were primarily involved in molecular functions including protein binding and transcriptional regulation. KEGG pathway enrichment analysis revealed that these genes were predominantly enriched in the following key pathways: the neurotrophin signaling pathway and the cAMP signaling pathway (Figure 6B). Among these, the cAMP signaling pathway exhibited the highest enrichment level and the largest gene count, followed by the neurotrophin signaling pathway. Collectively, these results implicate the associated genes in potentially playing critical roles in neuromodulation, metabolic regulation, and tumorigenesis.

## 4. Discussions

The “gut–liver axis” theory in modern medicine highlights the pivotal role of GM in the pathogenesis of PLC, establishing it as a critical interdisciplinary research focus [33]. Through the bidirectional regulatory mechanism of enterohepatic circulation, dysbiotic GM contributes to the pathological progression of PLC via multiple pathways, including metabolic dysregulation and immune imbalance. This biological link has been substantiated in multiple clinical studies [12, 34, 35]. This study integrated GWAS data on GM and PLC and leveraged MR analysis. It constitutes the first systematic identification of nine microbial features exhibiting genetically causal associations with the disease. Among these, five microbial groups, including the order Lactobacillales, demonstrated protective effects against PLC. Conversely, four groups, such as the class Mollicutes RF9, were associated with a significantly increased risk of PLC.

Notably, the mechanistic links between these microbial signatures and PLC have been corroborated across multiple research levels. For instance, *Ruminococcus*, a genus associated with increased cancer risk, can activate oncogenic pathways by promoting secondary bile acid production, while the protective order Lactobacillales inhibits endotoxin translocation to the liver by maintaining intestinal barrier integrity [36, 37]. Further studies demonstrate that fecal microbiota transplantation (FMT) from liver cancer patients significantly accelerates liver tumor formation in mice, and antibiotic intervention, by depleting protumorigenic microbiota, can partially block this tumorigenic process [38, 39]. These preclinical findings not only elucidate specific molecular mechanisms by which the GM modulates PLC but also provide biological plausibility supporting the causal inference in the present study. With the advent of novel technologies such as phage‐based targeted modulation, risk prediction models and precision intervention strategies for PLC based on gut microbial signatures are poised to transition from theoretical concepts to clinical practice.

Studies have shown that GM and their metabolites can participate in the pathological process of PLC through multiple molecular mechanisms [40, 41]. Gene functional annotation analysis revealed that the differentially expressed genes were significantly enriched in biological processes including positive regulation of the ERK1/ERK2 signaling pathway, posttranslational modifications such as deglutamylation, regulation of synaptic vesicle exocytosis, and transmembrane urea transport. At the metabolic pathway level [42], KEGG analysis further demonstrated that these genes are primarily implicated in activating neurotrophin signaling and cAMP signaling pathways. In addition to the cAMP signaling pathway and neurotrophic factor signaling pathway, the GM may be involved in the development of PLC through immune‐mediated pleiotropic pathways. Existing studies have confirmed that immune cell phenotypes are causally associated with the risk of digestive system tumors and diseases such as biliary tract cancer [15] and pancreatitis [43, 44], while gut microbiota dysregulation can regulate the hepatic immune microenvironment through the gut–liver axis, influencing the activation and function of immune cells. Notably, although direct experimental evidence establishing GM‐mediated regulation of these pathways remains elusive, multiple studies have confirmed significant correlations between the abundance of specific microbial taxa and the activation status of these pathways. Mechanistically, GM dysbiosis may lead to aberrant increases in metabolites such as endotoxins and secondary bile acids [45]. Upon entering the liver via the portal venous system, these substances activate TLR4 receptors and trigger downstream NF‐*κ*B signaling cascades, consequently inducing chronic inflammation and oxidative DNA damage [46]. Specific GM, such as the Mollicutes RF9 lineage and *Ruminococcus* species, may indirectly modulate the activation status of neurotrophic factor signaling pathways by regulating the secretion of metabolites associated with the TLR4 pathway [47, 48]. This interaction is primarily mediated through two mechanisms: Firstly, microbial metabolites can regulate immune cell functionality within the hepatic microenvironment. Secondly, these metabolites may modulate phosphorylation dynamics of key proteins in the cAMP signaling pathway via epigenetic modifications. Targeted modulation of these microbial communities significantly reduces the incidence of PLC in animal models, providing compelling albeit indirect evidence for the “GM‐signaling pathway‐PLC” causal axis [49].

Building upon contemporary antitumor approaches such as chemotherapy and targeted therapy, this study further explores TCM solutions for PLC through a “gene‐chemical component‐TCM” pathway. TCM demonstrates significant advantages in preventing PLC occurrence, recurrence, and metastasis, mitigating treatment‐related adverse effects and drug resistance, and improving disease control rates, objective response rates, and survival rates. Analysis of SNPs and their proximal genes associated with PLC within the CTD identified 142 representative chemical components, including doxorubicin, LPS, cisplatin, and furan. Cisplatin and doxorubicin serve as cornerstone chemotherapeutic agents for HCC. LPS is a recognized risk factor promoting PLC development, while furan derivatives have been shown to interfere with the metabolism of targeted agents used in HCC treatment, thereby compromising their efficacy. These findings collectively indicate that the identified chemical components play pivotal roles in HCC pathogenesis and progression. Consequently, we utilized this set of chemical components to predict potential TCM interventions.

This study predicts that TCMs, including *Panax ginseng* C. A. Meyer, Fructus *Schisandra chinensis*, Herba Hedyotis Diffusae, *Zingiber officinale* Rose., *Curcuma longa* L., Semen Ginkgo Bilobae, Cortex Cinnamomum Cassiae, and *Salvia miltiorrhiza*, are not only commonly used but also frequently employed in treating PLC and its complications. Pharmacological studies have confirmed the potential of these predicted Chinese medicinal interventions in preventing and treating PLC. Represented by *Panax ginseng* C. A. Meyer: These herbs can promote apoptosis in human hepatocellular carcinoma cells and induce cell cycle arrest by activating the c‐Jun N‐terminal kinase (JNK)/p38 MAPK signaling pathway via reactive oxygen species (ROS) mediation [50, 51]. Specifically, ginsenoside Rk3 directly targets the PI3K/Akt pathway, inhibiting AKT phosphorylation [52]. This downregulates downstream antiapoptotic factors and activates autophagy‐related proteins, leading to autophagic cell death in mouse liver cancer cells. Represented by Radix *Scutellaria baicalensis*: They inhibit the motility, adhesion, and invasive capacity of liver cancer cells by reducing matrix metalloproteinase 2 (MMP2) expression, increasing tissue inhibitor of metalloproteinase 2 (TIMP2), and modulating adhesion molecules [53]. The active constituent baicalin further suppresses the growth and metastatic potential of PLC cells and induces apoptosis by decreasing the expression of Bcl‐2, c‐Myc, cyclin D1, MMP‐9, and VEGFA while increasing Bax expression [54]. Represented by *Camellia sinensis* root [55]: Primarily through phenolic compounds like epigallocatechin gallate (EGCG), they exert antitumor effects by scavenging free radicals and activating the Nrf2 antioxidant pathway to mitigate oxidative stress damage. Concurrently, they suppress the NF‐*κ*B inflammatory pathway, reducing the release of proinflammatory cytokines such as TNF‐*α* and IL‐1*β*. Represented by *Salvia miltiorrhiza* [56]: These herbs significantly reduce PI3K p85 subunit expression and inhibit phosphorylation of AKT and mTOR, thereby blocking tumor cell survival signals. Additionally, they upregulate p21 expression via the p53 signaling pathway, inducing cell cycle arrest at the G0/G1 or G2/M phase and inhibiting liver cancer cell growth. Curcumin from *Curcuma longa* L. [57]: Curcumin interrupts the Wnt signaling pathway by binding to Dvl2 protein and hindering the membrane recruitment of Axin, thereby maintaining a functional *β*‐catenin destruction complex. This prevents nuclear translocation of *β*‐catenin and its binding to LEF/TCF transcription factors, leading to downregulation of target genes (c‐Myc, VEGF, and cyclin D1). Consequently, curcumin inhibits proliferation and induces apoptosis in PLC cells in a concentration‐dependent manner.

Numerous studies have highlighted the significant role of the GM in the pathogenesis and progression of PLC. However, findings from observational studies are susceptible to confounding factors. Given the unique advantage of MR in inferring causal effects, this study innovatively employed MR analysis to identify GM taxa exhibiting significant causal associations with PLC development. Building upon this, predicting potential TCM interventions via the proximal genes linked to MR IVs holds substantial implications for PLC prevention and treatment. This approach offers valuable insights and a reference framework for future research and development of novel TCM agents targeting PLC.

This MR study has the following limitations: Firstly, the horizontal pleiotropy of IVs is difficult to completely eliminate. Although adjusted by methods such as MR‐Egger, bias may still be introduced due to unrecognized pleiotropic SNPs. Secondly, the utilized GWAS data may have limitations in population representativeness, and differences in genetic backgrounds and phenotypic measurements across different cohorts could potentially impact the generalizability of the results.

## 5. Conclusion

Based on the predicted TCM, this MR study identified nine gut microbial taxa, including genus *Anaerotruncus*, genus *Barnesiella*, and genus *Catenibacterium*, which exhibit genetic causal associations with PLC development. These microbial taxa primarily mediate the pathogenesis of PLC through signaling pathways such as cAMP and ERK1/ERK2. Concurrently, potential TCM agents predicted to modulate these key signaling pathways via GM regulation, thereby influencing PLC progression, were identified. This provides valuable insights for exploring TCM prevention and treatment strategies for HCC from the GM perspective. Current research, both domestic and international, indicates that the mechanisms by which TCM regulates PLC occurrence through the GM involve not only alterations in gene or protein expression but also modulation of diverse host metabolic pathways. Consequently, incorporating metabolites as mediating variables in future studies could enhance the research framework. Given the prohibitively high cost of whole‐genome sequencing, the GWAS data utilized for the MR analysis in this study were primarily sourced from publicly available databases. Future research necessitates more in‐depth experimental investigations to elucidate and validate the biological mechanisms by which specific GM contributes to PLC pathogenesis and the regulatory role of TCM in this process.

## Author Contributions

Qingliang Chen, Lin Jin, and Suping Ding: conceptualization, methodology, software, and writing—original draft preparation. Lishan Ding, Liutong Zhang, and Shujuan Zuo: visualization, investigation, and writing—reviewing and editing. Wenle Fu and Pengchao Zhan: supervision, formal analysis, and writing—reviewing and editing. Qingliang Chen, Lin Jin, and Suping Ding had equal contributions to this work.

## Funding

This study was supported by the Medical Science and Technology Project of Henan Province (LHGJ20240648), Zhengzhou Medical and Health Field Science and Technology Innovation Guidance Project (2024YLZDJH056), Zhengzhou City Medical and Health Science and Technology Innovation Guidance Plan Project (2024YLZDJH044), and Henan Charity Federation Dao Jian Fund Scientific Research Project (szsyky24007).

## Disclosure

All authors have read and agreed to the published version of the manuscript.

## Ethics Statement

Ethical approval and consent to participate were waived by the Research Ethics Committee of Henan No. 3 Provincial People’s Hospital.

## Conflicts of Interest

The authors declare no conflicts of interest.

## Data Availability

The data used in this study are publicly available. The summary statistics of gut microbiota and PLC were obtained and extracted from the online public database (IEU OpenGWAS Project https://gwas.mrcieu.ac.uk/).
